# A Short Review on the Relationships Between Nephrolithiasis and Myocardial Infarction

**DOI:** 10.31661/gmj.v8i0.1289

**Published:** 2019-06-02

**Authors:** Hormoz Karami, Hadi Maleki, Maryam Baghbeheshti, Mostafa Hashemi, Mehrdad Rouzbeh, Mohammad Afkhami Ardakani

**Affiliations:** ^1^Department of Urology, Shahid Doctor Rahnemoon Hospital, Shahid Sadoughi University of Medical Sciences, Yazd, Iran; ^2^Student Research Committee, Yazd Cardiovascular research center, Afshar Hospital, Shahid Sadoughi University of Medical Sciences, Yazd, Iran; ^3^School of medicine, Student Research Committee, Shahid Sadoughi University of Medical Sciences, Yazd, Iran; ^4^Yazd Diabetes Research Center, Shahid Sadoughi University of Medical Sciences, Yazd, Iran

**Keywords:** Nephrolithiasis, Myocardial Infarction, Kidney Stone, Cardiovascular

## Abstract

The interaction between organs is a crucial part of modern medicine. As a very prerequisite to manage a disease, practitioners should have a full awareness of the related organs. Kidney and heart are two vital organs that are closely interconnected in various fields. These two organs have a lot of common risk factors for making a person unhealthy; therefore, if you prevent the disease in one of them, the other’s morbidity might be alleviated as well. Among them, nephrolithiasis and myocardial infarction (MI) have more risk factors in common, and both could be fatal. Also, these two diseases are important regarding the prevalence, incidence, and burden of disease. Some studies confirm the relationship between MI and nephrolithiasis; however, further researches are needed to discover the exact direction of their relationship. The present review aims to explain the mechanism of MI and nephrolithiasis; clarify the relationship between these two disease based on physiological, pathological, and clinical studies; and propose some solutions for the prevention and treatment of such diseases.

## Introduction

Nowadays, the prevalence of nephrolithiasis is rising in all countries because of the global warming phenomenon [1]. Noticeably, nephrolithiasis is more prevalent in countries located in hot and dry areas or those with more extensive deserts than other countries. Besides, cardiovascular diseases (CVDs) have an increasing prevalence accounting for 35% of the world’s annual mortality due to the lifestyle changes, pervasive tobacco consumption, increased rate of diabetes, and other risk factors [2, 3]. More interestingly, global warming alone can lead to an increase in CVDs [4]. Smoking as the important risk factor of CVDs is not only limited to a cigarette but also inhalation of polluted air containing multiple equivalents of cigarette smoke that can be easily inserted into the lungs, that is to say, mounting a risk onto a higher risk. To date, some studies have been conducted on various aspects of diseases. Nowadays, the bodily organs are examined separately owing to the progress of science, better recognition of organs, and branching of science; hence, specialists deal with each organ in a super-specialty manner and consult each other, if necessary. In the past, there was limited knowledge about the individual parts of the body but interconnection between organs. The issue of science islanding renders everyone a limited view of one’s specialty, giving rise to the remarkable relocation of our patients from a physician to the other. Acquiring information associated with the relationships among different organs can dramatically reduce the prevalence and incidence of various diseases, disabilities, and subsequent mortalities. Additionally, as vital organs are more important in the body, their interconnectedness should also be of the higher significance to us. For example, kidney is a vital organ which correlates with a lot of organs like bones [5-8], intestine [9-12], parathyroid [13-17], gallbladder [18-21], adrenal [22], thyroid [23], and heart (-1). Among the vital organs, the risk factors of cardiovascular and renal diseases are more common [24]. The associations of some diseases, such as CVDs and chronic renal failure, have been demonstrated in a variety of studies; however, there are conflicting reports on the relationship between myocardial infarction (MI) and nephrolithiasis. We reviewed the relationship between these two diseases using hitherto available studies to discover the effects of these two vital organs on one another, prevent common diseases of the heart and renal, and provide innovative solutions to cope with their related diseases. Indeed, the present study aims to achieve better understanding of the hypothesis representing the relationship between nephrolithiasis and MI and propose some clues to treat and manage a disease to prevent the other.

### Mechanisms of Nephrolithiasis

Although renal stone formation is one of the oldest human diseases, its mechanism is still unclear and there are many ambiguities in this regard [25, 26]. Nephrolithiasis has an extremely high prevalence, with a greater prevalence reported in the Western Hemisphere, e.g., 5-9% in Europe; 12% in Canada; and 13-15% in the United States, but 1-5% in the Eastern Hemisphere. In addition, recurrent nephrolithiasis has a considerably high rate throughout lifetime, such that it was reported about 10% in the first year, 35% up to 5 years, and 50% up to 10 years after the first stone formation [27]. The highest prevalence, albeit, was 20% in Saudi Arabia with a relapse rate of up to 50% during lifetime [28]. Depending on the composition, the most prevalent types of renal stone respectively include calcium oxalate [circa 75%), calcium hydroxyl phosphate (circa 50%), struvite (circa 10-20%), urate (circa 5%), cysteine (circa 1-2%), and the rest [29, 30]. There are various causes for the nephrolithiasis; however, the main cause is the super-saturation of stone-forming salts in urine. Other cases include decreased urine output; dropped pH; and high levels of calcium, oxalate, sodium, and urea, particularly in calcium oxalate stones formation [31]. Preventive substances for the stones contain organic citrate and magnesium, inorganic substances (nephrocalcine and osteopontin), and urinary prothrombin fragment [32, 33]. In fact, the underlying bases for stone formation are urinary inorganic super-saturation and lack of crystallization inhibitors [34, 35]. A series of proteins (e.g., Tamm–Horsfall protein) inhibits the growth and interconnection of calcium oxalate stones. It should be noted that such proteins play a role in preventing crystallization; however, once immobilized on the surface, they provide a substrate for stone formation. The pathogenesis of nephrolithiasis involves several stages of nucleation, crystal growth, crystal attachment, crystal clinging, and finally, the formation of stones on these crystals [36, 37]. The first stage in the renal stone formation is nucleation, i.e., the conversion of soluble materials into solids. The primary nucleus has a lattice state. Epithelial cells, urine casts, red globules, and other crystals can serve as nuclei in urine [38]. The matrix ​​actively contributes to the stone formation. The matrix component comprises 2-3% of net stone weight made up of urine macromolecules. These macromolecules contain 64% protein, 9.6% non-amino sugars, 5% hexamine (e.g., glucosamine), and 10% polarized water, lipids, and ultimately minerals. These urinary molecules lead to leftovers of urinary crystals in the collecting ducts, and consequently to the formation of stones. Then, the crystal grows in the matrix of nucleation and comes in contact with supersaturated urine [26, 39-42]. The interaction between the crystals and the cells of renal epithelial tubules, including clinging or endocytosis of the crystal, is the most prominent factor in the formation of renal stones. Damages to renal tubular cells can pave the ground for the formation of calcium stones by providing materials for the nucleation. According to the recent studies on the stone-forming people, chemical changes in urine may lead to epithelial damage. Intra-renal crystals stimulate tumor necrosis factor receptors, thereby, result in inflammation and apoptosis of renal cells [43-45]. The intra-tubular crystals are bonded to membranes of epithelial cell tubules aided by a group of binding molecules [46, 47]. It is the attachment site of nuclear crystal that forms an intra-tubular plaque resulting in nephron occlusion and atrophy [48-50]. Based on the normal secretion of oxalate and morphology of the nephrons, the particle should not reach a size to tangle up in the nephrons. The assumption of damages to the urothelium leads to the tangling of the crystal in the nephrons giving rise to nucleation followed by cellular damage and completion of stone formation cycle [30].

The intra- and extra-cellular formation events of calcium oxalate stones include initiation of the urinary super-saturation with oxalate or calcium in the setting of hyperoxaluria and hypercalciuria. Subsequently, oxidative stress brings about cellular apoptosis or necrosis, causing cellular damage and membrane ruptures. Initially, expression of crystalline-binding molecules (including osteopontin, hyaluronic acid, sialic acid, and CD44) increases, and consequently, these molecules are exposed and the crystals are attached to the cellular membrane. The crystals are then transferred to the interstitial tissue causing inflammation and chemoattractant protein I. Alternatively, cellular damage facilitates the nucleation and grows on the crystal nucleus, followed by the juxtaposition of crystals. Meanwhile, the crystal and the cell interact with each other, the crystals are connected, and eventually, the stone is formed. Thus far, various mechanisms have been proposed for the nephrolithiasis, of which four mechanisms are discussed below [46, 51]. The first mechanism occurs in the formation of calcium oxalate stones on Randall’s plaque in people with hypercalciuria. The second mechanism as the most common phenotype is the growth of calculi at the end of the papillary (Bellini) ducts. The third mechanism arises in people who make cysteine calculi consisting of microliths formed in the collecting ducts of the renal medulla. The last mechanism includes stone formation in the case of cysteine ​​calculi, which is the development of small, oval, and yellow calculi in the free fluid inside the calyx. According to these mechanisms, four sites for the start of calculus formation include Randall’s plaques, Bellini ducts, inner medullary collecting ducts, and the calyx fluid [52]. Randall’s plaques are made up of hydroxyapatite, the theory of which was first put forth in 1938. The first site for the formation of Randall’s plaques is the basal membrane of the thin loop of Henle (LoH)[53], which is a modification of the lamina matrix in the form of small circles. The role of this part as the first site of stone formation can be explained by the compressive effect of fluid movement inside the lumen and interstitial tissue. In animals, this part of LoH has been proved to be composed of ultra-saturated calcium phosphate compounds. While in humans the ionic composition of this part is yet unknown. This part of LoH is naturally impermeable to calcium, calcium is expelled from the proximal tubule due to the super-saturation of LoH content with calcium in people having idiopathic hypercalciuria, resulting in high level of calcium exposure during the daytime [54].The nucleus of this plaque is separately located in the interstitial tissue, which is interconnected in the basal membrane, near to which hyaline crystals deposit. These plaques are situated between the tubules and the vessels. Afterward, the urothelium is pushed out and white plaques are seen at the end of the urinary calyces. In fact, the mechanism for the formation of calcium oxalate stones in individuals with idiopathic hypercalciuria involves the following steps. First, the core of Randall’s plaques in the basal membrane of the thin LoH is made as the multi-layer circles of matrix and crystal. Then interstitial plaques are formed on collagen type I in the interstitial space. The urothelium then thrusts out these plaques and comes in contact with urine. Then, urinary proteins (e.g., osteopontin and Tamm–Horsfall) are placed on it and covered by hydroxyapatite crystals, followed by the coverage of calcium oxalate crystals [55, 56].

The mechanism of forming calcium oxalate stones on the plaques in hyperparathyroidism and patients with an ileostomy and those with a resected small intestine is different from that of idiopathic people. The second mechanism is the formation of calculi in the creation of a plaque in Bellini ducts. First, diluted Bellini ducts are filled with crystals, which sometimes grow into the urinary lumen, and then hyaluronic acid and brushite (CaHPO4 2H2O) grow on this plaque [57]. In fact, individuals with brushite stones, there is fossa at the tip of the papilla as the opening sites of the Bellini ducts, which not only are dilated, filled with crystals, and embossed in urethra but also pave the way for the formation of stones [58]. Ileostomy, primary hyperparathyroidism, distal renal tubular acidosis (RTA), resection of the small intestine, cystinuria, and glycosuria are the risk factors for formation renal stones with this mechanism [59, 60]. Apart from the stone formation through this mechanism, the presence of the plaque also may cause damages to the kidney papilla, resulting in the complete destruction of the tubule epithelium rendering the crystal attached to the basal membrane. The collecting ducts’ diameters reach approximately 20 times larger than the normal size and the surrounding interstitial tissue turns into fibrosis. In some patients, these plaques extend to the inner medullary ducts. These cortex changes are not observed in people with calcium stones, but it reported in brushite and hydroxyapatite stones. The phenotypes of stones produced by this mechanism are abundant, and hydroxyapatite and brushite stones are always made with this mechanism. The third mechanism of renal stone formation involves yellow mineral deposition inside the lumen of the medullary collecting ducts underneath the urothelium. The deposition of these materials can vary widely from spoke-like to a small area. After its formation and growth in the collecting duct, the plaque is separated, grows within the calyx, and is excreted thereafter. The fourth mechanism includes stone formation in the free liquid, which is most commonly seen in cysteine ​​calculi. In fact, cysteine grits of 1-2 mm are made in the distal medullary ducts, which are round and small and do not cling to the epithelium of these ducts. In this mechanism, the stones fill the calyx with no visible sites of adhesion to the epithelium. It observed in cases of calcium oxalate stones, primary hyperoxaluria, brushite, hydroxyapatite, gastrointestinal bypass surgery, and all cysteine stones [61, 62]. In other words, there are two stone precursor sites in the kidney papilla. Both sites with 1 and 2 cores are meant for crystalline deposition and stone formation. One is Randall’s plaque (Type I precursor site) in which calcium, phosphorus, and calcium carbonate deposit in the sub-endothelium interstitial space; emerge from the kidney papilla; and erode the surface of the papilla. In Type II precursor site or Randall’s plaque, salts, debris, and epithelial cell necrosis lead to the plaque formation in the setting of ​​super-saturation, urine flow, and the internal size of the ducts [63]. Calcium phosphate stones in primary hyperparathyroidism, calcium oxalate stones in primary hyperoxaluria, stones in the field of slimming surgery, cysteine, and brushite, as well as some cases of idiopathic calcium oxalate stones, attach to Randall’s plaque, leading to inflammation and damage to renal tubules [64]. Both CVDs and nephrolithiasis occur on the matrix accumulated with proteins and oxidative lipids in the renal tissue and vascular walls. CVDs and nephrolithiasis are both among multifactorial cases, environmental interaction, and genetic factors. Reactive oxygen spices are the linking agents of nephrolithiasis and CVDs (-2)[65-67].

### Mechanisms of MI

The activity of myocytes and their normal function are highly dependent on the oxidative metabolism. In addition to this dependence, myocytes are very sensitive to O2 variations. Any factor that reduces cellular O2 levels can lead to the apoptosis and necrosis of myocytes. Coronary obstruction as the most common cause of O2 reduction brings about energy starvation and ultimately myocardium necrosis [68]. The detachment of atherosclerotic plaque and erosion of coronary endothelium are responsible for most MI. If one has a stable plaque, it will cause angina when more than 70% of the vessel’s lumen is blocked by the plaque. Plaque rupture, the release of thrombogenic factors, and initiation of the coagulation cascade eventually lead to coronary occlusion and MI [69, 70]. The definition of MI has undergone some changes over the years because the use of the term MI can impose a significant burden regarding economic, legal, scientific, and economic aspects. Nowadays, the diagnosis of MI was made based on patients’’ symptoms, changes in the electrocardiogram, and cardiac enzymes [71].

### Relationships Between Nephrolithiasis and MI

Reduced glomerular filtration rate (GFR) and albuminuria are associated with coronary disease. In addition, nephrolithiasis is known as a risk factor for MI. As a result, an increased risk of MI following a nephrolithiasis-induced chronic kidney disease has continuously been a matter of concern [72]. Coronary calcification is known as one of the significant factors in CVDs. Notably, 80% of nephrolithiasis have a calcium component. A consensus currently exists on hyperuricemia as a risk factor for the formation of uric acid stones, as well as for coronary diseases [72-74]. There is some evidence that may justify the relationship between nephrolithiasis and MI. For example, smoking, caffeine, and aging independently increase nephrolithiasis and also CVDs. Also, such risk factors as subclinical atherosclerosis, hypertension, diabetes and metabolic syndrome are the intersection between cardiovascular events and nephrolithiasis [15, 75]. Nutritional factors (e.g., lack of calcium intake) are directly related to the increased likelihood of nephrolithiasis and the incidence of hypertension as a major risk factor for CVDs. There is also a need for cardiac revascularization in people who have had a history of nephrolithiasis [74-77].

## Discussion

Given the strong associations between various bodily organs and vital organs, including the heart and kidneys, as well as the considerable sharing of risk factors among the two organs, it is apparently possible to prevent other ailments by preventing diseases related to these two organs. For example, high water intake can lead to better circulation, reduce coronary spasm and MI, and prevent nephrolithiasis according to the mechanisms described above. Less salt intake results in a decreased salt sedimentation in the kidneys and can provide cardiovascular health by lowering blood pressure. Adequate dairy consumption through adequate calcium supply can also prevent hypertension and the need for cardiac revascularization along with the lower rate of nephrolithiasis. Regular exercise can contribute to improved GFR and increased pre-renal volume by better oxygenation of myocardia and enhanced circulation leading to prevention of nephrolithiasis. The role of nephrolithiasis treatment on the possibility of reducing cardiovascular disorders is a fascinating subject that needs further studies in the future. Due to ever increasing advances in science and detailing of various sciences, especially in the medical field, one should not overlook the fact that the wide interrelations of organs as the probable treatment of many diseases today entails a strong group work. Furthermore, when it becomes feasible to discover the common main cause of diseases, considerable steps can be taken in the prevention and treatment of common organ diseases, which also lowers the costs for the society.

## Conclusion

What follows from this study implies that there are many relationships between the heart and kidneys in terms of both diseases and risk factors. Among the diseases, what discussed more in this study was the relationship between nephrolithiasis and MI. However, the issues like ‘which organ has a more dominant influence on the other’ or ‘diseases of which organ are the leading cause of the other one’ are debatable issues and require more extensive studies in this field.

## Conflict of Interest

The authors declare no conflict of interests.

**Figure 1:**
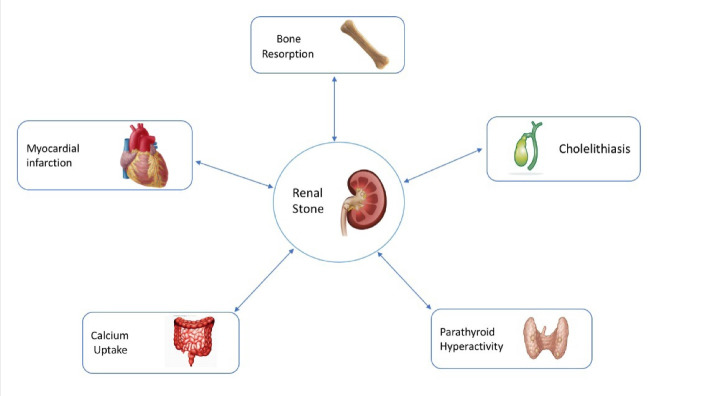
Kidney and main organs which related to nephrolithiasis

**Figure 2:**
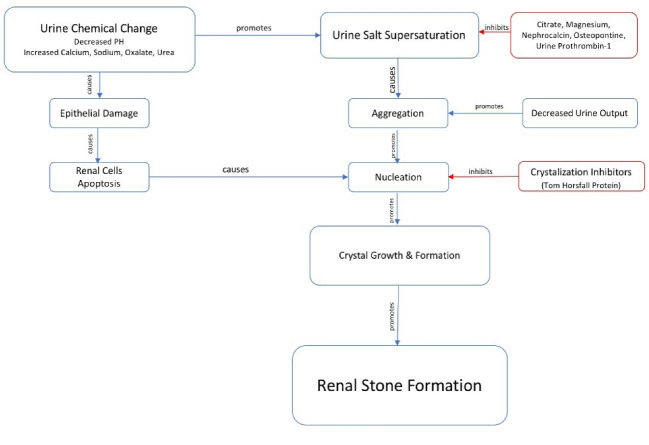
Concept map of renal stone formation
